# Detection of Markers in Green Beans and Roasted Beans of Kalosi-Enrekang Arabica Coffee with Different Postharvest Processing Using LC-MS/MS

**DOI:** 10.1155/2023/6696808

**Published:** 2023-03-23

**Authors:** Yulianti Yulianti, Dede Robiatul Adawiyah, Dian Herawati, Dias Indrasti, Nuri Andarwulan

**Affiliations:** ^1^Department of Food Science and Technology, Faculty of Agricultural Engineering and Technology, IPB University, IPB Dramaga Campus, Bogor 16680, Indonesia; ^2^South-East Asia Food & Agricultural Science and Technology (SEAFAST) Center, IPB University, IPB Dramaga Campus, Bogor 16680, Indonesia; ^3^Department of Agricultural Technology, Faculty of Agriculture, Gorontalo University, Gorontalo 96211, Indonesia

## Abstract

Our study is aimed at evaluating the effect of postharvest processing (natural, honey, and fully washed) on the compounds profile in green beans and roasted beans of Kalosi-Enrekang Arabica coffee and determining the marker compounds for each process. These beans were extracted using boiling water, and the extract was analyzed using LC-MS/MS. The results of this work confirmed the significant impact of postharvest processing on compounds in the coffee beans, and each process has a marker compound. Green beans by natural processing have 3 marker compounds, honey processing has 6 marker compounds, and fully washed processing has 2 marker compounds. Meanwhile, roasted beans by natural processing have 4 marker compounds, honey processing has 5 marker compounds, and fully washed processing has 7 marker compounds. In addition, our research identified caffeoyl tyrosine in green beans from natural and honey processing, which was previously only identified in Robusta coffee. These marker compounds can differentiate postharvest processing (natural, honey, and fully washed). These results can also help understand the effect of postharvest processing on the chemical composition of green and roasted beans.

## 1. Introduction

Quality is an essential parameter in the international trade of coffee. It is affected by 40% of geographical factors and 60% of postharvest processes (40% of primary processes and 20% of secondary processes) [1]. Postharvest processing markedly determines the coffee quality [2]. In Indonesia, postharvest coffee processing is performed by two popular methods: natural and fully washed. However, nowadays, honey processing has been adopted as a new processing procedure applied by some farmers. Each has different stages of processing. The natural process produced dried coffee cherries without removing their exocarp. In contrast, in the fully washed process, the cherries were dried after the removal of skin, soaking in water (fermented), and removal of mucilage (washed) [1, 3]. According to Sanz-Uribe et al. [4], the honey process was performed by skin depulping, fermentation, and drying. It is called honey processing because the mucilage is dried together with the beans, and after drying, it produces the aroma of honey and sugar [5].

The chemical content of green beans is influenced by postharvest processing and will affect the final quality of brewed coffee [6–8]. Besides the coffee postharvest processing, the presence of drought stress can affect the senses of coffee. This difference occurs due to metabolic processes that cause changes in the chemical composition of coffee beans [9]. The biochemical reactions occurring in different compounds during fermentation also contribute to coffee's chemical composition and taste [4, 10]. According to Tsukui et al. [11], the compounds for distinguishing green beans processed by pulped natural and natural processing were glycosylated atractyligenins and carboxyatractyligenins. This study used three postharvest processes: natural, honey, and fully washed. Therefore, further research is needed to identify marker compounds in various postharvest processing.

In addition to postharvest processing, the roasting process can influence the chemical composition and flavor of the coffee. During the roasting process, two mechanisms occur, including heat transmission and temperature profile, and finally, they impact roasted coffee beans' physical and chemical qualities [5, 12]. Moreover, many complex chemical reactions that occur during roasting because the formation of compounds that affects the taste of brewed coffee [13]. Such reactions include Maillard and Strecker degradation, hydrolysis, and pyrolysis, which are responsible for coffee's color and aroma [14]. Furthermore, the roasting stage decreased the moisture content, which affected the coffee beans' characteristics [15]. The profile of volatile compounds formed during roasting has been widely studied [16–21]. However, the compounds profile in roasted coffee beans processed by different postharvest processing is still limited, especially in identifying the presence of marker compounds inter-postharvest processing. Therefore, this study is aimed at evaluating the effect of different postharvest processing on green and roasted bean compounds profiles. In addition, this study investigated the marker compounds that distinguished postharvest processing in green and roasted beans. This study applied an untargeted analytical method and could assess the presence of mixing or adulteration in commercial coffee beans.

## 2. Materials and Methods

### 2.1. Materials

Materials are Kalosi-Enrekang Arabica coffee cherries, formic acid 98-100% for analysis, methanol, and water for chromatography (LC-MS grade) (Merck, Darmstadt, Germany).

### 2.2. Sample Preparation

**Figure 1:**
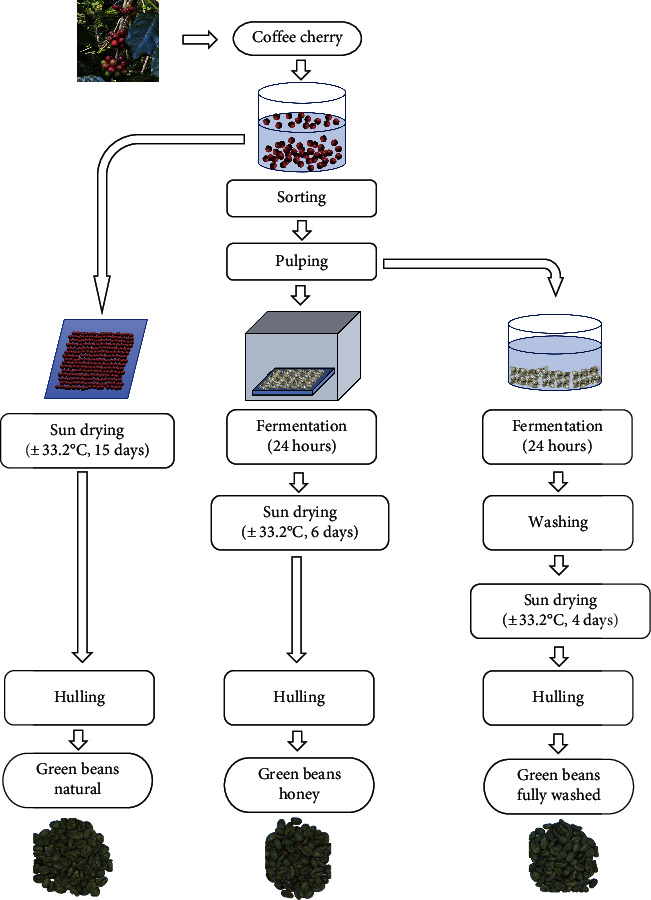
Postharvest processing applied to Kalosi-Enrekang Arabica coffee. In the honey process, coffee beans were fermented in a room not exposed to the sun. In the fully washed process, coffee beans were fermented by soaking in water.

Kalosi-Enrekang Arabica coffee cherries were harvested in May 2021 and treated at different processing conditions (i.e., natural, honey, and fully washed). Before processing, coffee cherries are sorted manually by soaking them in water and removing floating cherries (defective). The stages of postharvest processing can be seen in Figure 1.

**Figure 2:**
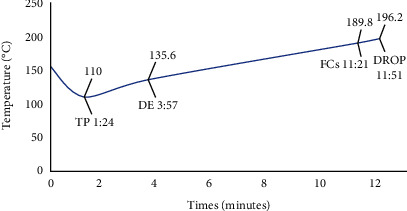
Temperature profile during roasting of Kalosi-Enrekang Arabica coffee. TP: turning point; DE: development; FCs: first crack; DROP: coffee beans dropped from the drum.

Green beans were roasted using a roasting machine from PT Kemenady Industri Mandiri (IKRI, Jember, Indonesia). The initial temperature was 155°C, and the final temperature was 196°C after 12 minutes. Roasted beans are classified as medium degree (lightness: 36-37, Konica Minolta CR400, Konica Minolta Inc., Japan). The roasting time and temperature can be seen in Figure 2.

Green and roasted beans were ground using a coffee grinder (Gemilai crm905, China). Before being ground, the green beans were first added to liquid nitrogen. Green bean and roasted bean extraction complied with the method of Herawati et al. [22]. Powdered green beans and roasted beans (5 g) were dissolved in 100 mL of boiling distilled water. The solution was then heated to 90°C, stirred using a magnetic stirrer for 1 minute, cooled in an ice bath for 2 minutes, and stirred continuously. The solution was filtered using Whatman filter paper no. 1 to obtain coffee filtrate, then stored in the freezer at -22°C for analysis.

### 2.3. Profiling and Analysis of Chemical Compounds

The determination of compounds followed the method of Herawati et al. [22] using UHPLC Q Orbitrap HRMS (Thermo Fisher Scientific, Bremen, Germany). Filtrates of green beans and roasted beans were diluted with methanol grade LC-MS (1 : 1, *v*/*v*) and filtered through a 0.22 *μ*m PTFE membrane (Merck, KGaA, Darmstadt, Germany). The 5 *μ*L of the sample was injected into the Accucore C18+column (100 × 2.1 mm, 1.5 *μ*m) (Thermo Fisher Scientific, Bremen, Germany) at 40°C. The mobile phase was methanol LC-MS (a) and 0.05% formic acid in water LC-MS (b); they were set at a flow rate of 0.2 mL/min, and the gradient elutions are 0 min (5% A), 0-17 min (90% A), 17-20 min (90% A), 20-23 min (5% A), and 23-30 min (5% A). Ionization modes are used in electrospray ionization (ESI) of positive and negative by mass Q Orbitrap analysis. Analysis was carried out at *m*/*z* 70-900 accompanied by UV detection at wavelengths 220, 265, 272, and 320 nm.

Identification of compounds was carried out by analyzing raw data (chromatograms) using Compound Discoverer 3.2 software (Thermo Fisher Scientific) and using online libraries (FoodDB, HMDB, NIST, Pubchem, and MzCloud). The MS^2^ suitability of the identified compounds was analyzed using the ThermoXcalibur 4.2 mass spectral library software (Thermo Fisher Scientific). The identified compounds were selected using Annot. DeltaMass <5 ppm. Quantifying the compound was done by comparing the compound's area to the total area of the eluted compound (% relative).

### 2.4. Data Analysis

Quantitative data analysis (% relative) was performed using an ANOVA with a single factor followed by Duncan's test (significantly different at *P* < 0.05). The T-test was carried out between green beans and roasted beans (*P* < 0.05) using Microsoft Excel 2019 software (One Microsoft Way, Redmond, USA). Multivariate analysis was designed to depict the presence of marker compounds in green and roasted beans using OPLS-DA (orthogonal projection to latent structures-discriminant analysis) modeling using the SIMCA 14.1 software. OPLS-DA is an unsupervised pattern recognition method that provides maximum covariance between the measured data (*X*) and the response variable (*Y*). The OPLS-DA model is considered to have a membership probability of 95% confidence level, and observations <5% are considered outliers. The overall predictive ability of the model was assessed by the cumulative Q^2^ representing the fraction of *Y* variation that can be predicted by the model [23]. Compound classes are referred to at https://foodb.ca.

## 3. Results and Discussion

### 3.1. Profile of Compound Classes in Green Beans and Roasted Beans

Identification of compounds using LC-MS/MS successfully identified 18 compound classes in green beans and 20 compound classes in roasted beans (Supplementary materials, Table 1). The compound classes in green beans dominantly belonged to quinic acid, alkaloids, amino acids, and coumarins (Table 1). The concentration of quinic acid and derivatives was affected by postharvest processing (*P* < 0.05). This compound class was more concentrated in natural processing than in fully washed and the honey process. This is obviously understood since natural processing is the most straightforward procedure where mucilage and pulp containing high carbohydrates are still attached to the beans. Drying causes carbohydrates to degrade [24]. The degraded forms of them are substrates for the synthesis of quinic acid and its derivatives. Quinic acid and derivatives are produced from the shikimate pathway using substrates from carbohydrate metabolism [25]. Coffee beans have the longest fermentation time in natural processing compared to fully washed and honey processing [26]. During drying, coffee is still metabolically active in response to abiotic pressure, anoxic conditions, and stress [27]. Long drying causes the chemical components of the pulp to experience translocation to the coffee beans [28].

The concentration of quinic acid and derivatives was 20.46-23.76% in green beans and decreased up to 10.24-10.95% after roasting (Table 1) since they experienced degradation and hydrolysis into new compounds. According to Lee et al. [29] and Yeretzian et al. [30], phenolic compounds, such as chlorogenic acid (CGA), were hydrolyzed into hydroxycinnamic acid derivatives. Meanwhile, hydroxycinnamic acids such as ferulic acid are decarboxylated and produce volatile phenolic compounds such as guaiacol, p-vinylguaiacol, and phenols. The concentration of CGA and its derivatives declines because the covalent bonds between carbons are broken down and isomerized in the early stages of roasting. In the next phase, it undergoes epimerization and lactonization [31].

In addition, an amino acid is found dominantly in green beans, and its amount is also influenced by postharvest processing (*P* < 0.05). The highest concentration of amino acids was observed in green beans prepared by a natural process, while the fully washed process resulted in the lowest (Table 1). The level of amino acids was markedly affected by the fermentation process. According to Lee et al. [32], a decrease in the concentration of amino acids resulted from oxidative deamination during fermentation, indicated by an increased concentration of ammonia. According to Lee et al. [33], fermentation causes a change in the modulation of the amino acid profile in green beans. In addition to the fermentation process, drying also affects the amino acid composition of green beans. de Melo Pereira et al. [7] found the accumulation of *γ*-aminobutyric acid in coffee beans from the natural process due to drought stress after prolonged heat exposure.

During the roasting process, the concentration of amino acids decreases (Table 1). This is because the amino acid is used as a substrate for the Maillard reaction during the roasting process. According to Lee et al. [29] and Yeretzian et al. [30], green bean components, such as sugars, proteins, amino acids, and phenolics, are the main precursors of aroma formation. The reactions that occur during roasting (pyrolysis, caramelization, and the Maillard reaction) affect the composition of the beans. Furthermore, the roasting process induced the fragmentation of proteins to form amino acids, leading to considerable changes in the protein profile [29].

Alkaloids in green beans are also affected by postharvest processing. Alkaloids are highest in fully washed processing and lowest in natural processing. The high concentration of alkaloids in fully washed processed is because the content of caffeine and trigonelline, which are the dominant compounds in the alkaloid group, can be maintained. Concentrations of caffeine and trigonelline in fully washed processed were 17.148% and 12.014%, while in natural processing, they were 15.839% and 10.858% (Supplementary materials, Table 1). Both of these compounds are not lost during the washing process. In addition, the drying time for fully washed processed is shorter than honey and natural processed, so caffeine and trigonelline are not further degraded. Caffeine has moderate solubility in water but is hydrophobic enough to pass through biological membranes [34]. A longer time of sun-drying process in natural processing raised the degradation of caffeine [3]. Duarte et al. [35] reported that the process of lixiviation and heat-induced degradation promoted the differences in trigonelline content in differently processed green beans. Mehari et al. [36] argued that the loss of high solubility compounds in water due to washing did not affect trigonelline concentrations in fully washed processing.

The roasting process also reduced the concentration of alkaloids from 26.92-29.38% in green beans to 24.14-27.04% in roasted beans (Table 1). However, the decrease in alkaloid concentration was not as much as the decrease in quinic acid and derivatives. This can be clearly explained that caffeine is present in the highest proportion compared to other alkaloids and is stable at high temperatures [37]. Unlike caffeine, trigonelline is easily degraded due to heat. Trigonelline is a precursor compound for the formation of taste and aroma in coffee. Trigonelline is unstable at high temperatures and easily degraded during roasting [38]. This compound contributes to the formation of furans, pyrazine, alkyl-pyridines, and pyrroles. The pyrolysis reaction causes trigonelline to form nicotinic acid and N-methylpyridinium [5, 13, 39, 40].

The coumarins acid and derivate classes decreased, while cinnamic acid increased during roasting. Both compounds resulted from the degradation of chlorogenic acid [5]. Other components identified in green beans were organoheterocyclic, organic acid, lipid, phenylpropanoid, carbohydrates, phenolic acids, benzenoid, carboxylic acids and derivatives, organonitrogen compounds, purine nucleotides, purines and purine derivatives, pyrimidine nucleosides, and keto acids and derivatives (Table 1). The different types of microorganisms involved in the fermentation process in each postharvest processing process produce different chemical compositions in green beans. Microorganisms result in sugar pectinase producing ethanol, lactic, acetic, butyric acid, and other carboxylic acids derived [41]. Acid compounds in fully washed processing are produced from lactic acid and acetic acid, which are microbiologically induced LAB (*Lactococcus*, *Leuconostoc*, and *Weissella*) present in mucilage. In the fully washed process, sucrose content perpetually decreased as carbohydrate was used in the glycolysis. The loss of some compounds is associated with fermentation and soaking processes, where endogenous metabolism and exosmosis occur. During the drying process, drought stress occurs, and it changes the concentration of specific compounds. The period of soaking, fermentation, and drying was reported to affect the composition of green beans [24].

The compound class identified after the roasting process included the carbonyl compound, pyrimidines and their derivatives, organooxygen compounds, and organosulfur compounds. These components are formed due to the reaction that occurs during roasting. Carbonyl compounds, organooxygen compounds, and organosulfur compounds are formed through the Maillard reaction with simple sugars and amino acids. The difference in concentration between postharvest processing depends on the concentration of precursors in the green beans. Reactions that occur during roasting (pyrolysis, caramelization, and Maillard reactions) lead to the formation of new compounds or components [42]. The chemical composition of green beans acts as the precursor of new compounds in roasted beans. A decrease in the concentration of the dominant compounds during roasting characterizes their presence. The formation of compounds in the roasting process is influenced by the composition of green beans [30, 43].

### 3.2. Marker Compounds in Green Beans and Roasted Beans

**Figure 3:**
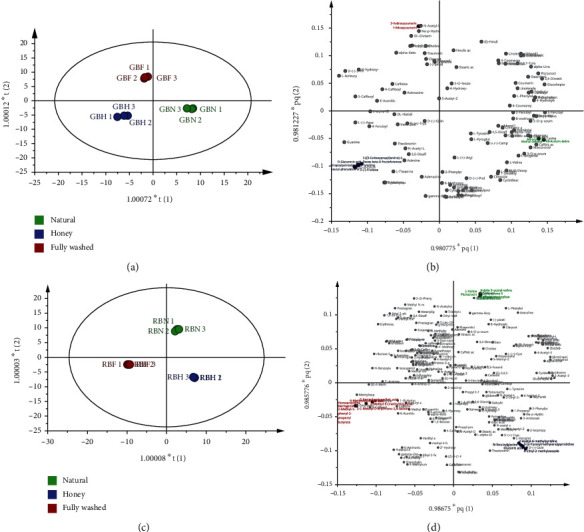
OPLS-DA compounds profiles of green beans (a, b) and roasted beans (c, d) of Kalosi-Enrekang Arabica coffee with different postharvest processing. Score plot (a, c) and loading plot (b, d). GBN: green beans natural process; GBH: green beans honey process; GBF: green beans fully washed process; RBN: roasted beans natural process; RBH: roasted beans honey process; RBF: roasted beans fully washed process.

Compounds were identified in green beans with more than 100 compounds and in roasted beans with more than 200 compounds (Supplementary materials, Table 1). Figures 3(a) and 3(b)) showed that the postharvest processing process had a significant effect on green beans metabolites (*P* < 0.05), and modeling with OPLS-DA was declared reliable and valid with a value of *Q*^2^: 0.938; *R*^2^*Y*: 1; *R*^2^: 0.994; and *R*^2^*X*: 0.767. Kulapichitr et al. [44] reported that postharvest processing remarkably contributed to the chemical composition of green beans. Similarly, roasted beans' chemical composition depended on postharvest processing (Figures 3(c) and 3(d)). This can be seen in the values of *Q*^2^: 0.951, *R*^2^*Y*: 1, *R*^2^: 0.999, *R*^2^*X*: 0.875, and *P* < 0.05. Worley and Powers [45] argued that OPLS-DA could be a meaningful modeling tool in metabolomics, enabling the identification of metabolite changes with separation between experimental groups. OPLS-DA modeling was used to identify correlations between experimental groups using metabolites [23]. Moreover, Guijarro-Díez et al. [46] mentioned that OPLS-DA could distinguish between groups and obtain significantly discriminatory compounds. The OPLS-DA model was declared reliable, excellent, and significant with a small *P* value, an *R*^2^ close to 1, and an acceptable *Q*^2^ value of ≥0.4 (the maximum value of *Q* is 1) [45, 47, 48]. Reliable OPLS-DA modeling was evaluated by the CV-ANOVA (CULTIVAR-ANOVA) analysis with a significance model of value *P* < 0.05 [49].

OPLS-DA modeling shows that green beans and roasted beans (Figure 3) from different postharvest processing are separate, and each processing demonstrates a marker compound. For validation, VIP (variable importance for the projection) predictive with a VIP value of *>*1.00 was used (Supplementary materials, Figure 1). Compounds have a VIP value of >1.00 important role in differentiating green beans and roasted beans between postharvest processing. In addition, compounds that are predicted to be interprocessing postharvest are only detected in one postharvest processing, both in green and roasted beans (Supplementary materials, Table 1). According to Zeiss et al. [50], VIP was applied to validate, enabling avoidance of deviations in selecting plot variables. A variable score of VIP >1.00 is significant for comparing two or more groups.

In this work, the peptide is predicted as a marker in green beans (Table 2). In the natural processed green beans, ala-pro is identified as a marker. While in the honey process, leucyl-phenylalanine is noted as a marker. According to Ludwig et al. [51]^,^ the postharvest process of coffee affected the composition of peptides due to the different enzyme activities in each postharvest processing. Peptides present in green beans contribute to the formation of flavor during roasting. Methyl caffeate and N6-Acetyl-L-lysine are also predicted to be marker compounds in the natural processed green beans (Table 2). Methyl caffeate is a compound produced from the phenylpropanoid pathway, in which caffeic acid compounds are esterified [52]. Whereas, N6-Acetyl-L-lysine belongs to the class of amino acids produced from the amino acid lysine catabolized by acetyl-CoA involving L-lysine N6-acetyltransferase (yeasts) [53].

Green beans from the honey process are estimated to possess a marker compound, namely sinapoylputrescine (Table 2). It is derived from the interaction of acyl donor sinapoyl CoA with acyl acceptor putrescine, specifically distinguished by enzymatic activity [54]. This compound is formed due to drought stress [50]. Moreover, 1-[(3-Carboxypropyl)amino]-1-deoxy-beta-D-fructofuranose is regarded as a marker in green beans in honey processing. This compound is also found in *Pangium edule* seed extract and has antifungal properties [55]. Tyramine, a marker compound in honey processing, is formed by the amino acid tyrosine, which releases the carboxyl group with the aid of the enzyme tyrosine decarboxylase (TDC) [56]. D-glucuronic acid and D-fructose are also predicted to be marker compounds in honey processing. Bruyn et al. [24] argued that D-glucuronic acid and fructose decreased during fermentation and soaking. In longer fermentation and soaking, they might disappear. Fermentation of beans by soaking them in water for 24 hours and washing after the fermentation was evidenced able to remove glucuronic acid and fructose compounds. At the beginning of drying, D-glucuronic acid increased, then it decreased with a longer drying time. These two compounds are predicted to be identified in green beans prepared by the honey process. The honey processing involved fermentation in the open space, but the beans were not exposed to direct sunlight. Furthermore, in honey processing, the washing process was not carried out after the fermentation process. Thus, both compounds can be maintained. In addition, in honey processing, the drying process period is shorter than the natural process, leading to maintaining compound stability.

**Figure 4:**
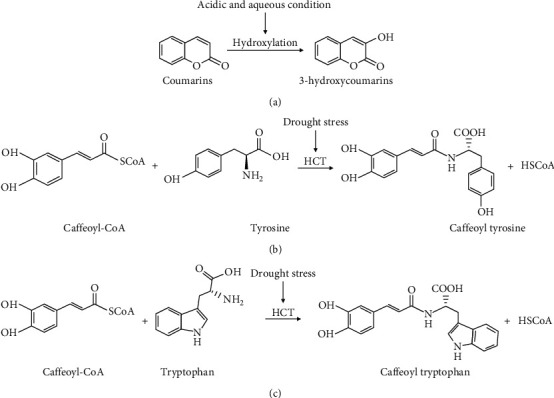
Scheme of formation of 3-hydroxycoumarins (a), caffeoyl tyrosine (b), and caffeoyl tryptophan (c). HCT: hydroxycinnamoyl transferase [57, 58, 63].

In this work, we reported marker compounds in the fully washed processed green beans, including 3-hydroxycoumarins and 7-ethoxycoumarin. 3-Hydroxycoumarins are produced from coumarins through a hydroxylation mechanism under acidic conditions (Figure 4(a)) [57, 58] and under aqueous conditions [59]. Fully washed processing was performed by fermenting coffee beans by soaking them in water. The fermentation condition is acidic (pH 4). One of the compounds which are only identified by natural process and honey-processed green beans is caffeoyl tyrosine (Table 2). Based on our investigation, this compound is only identified in Robusta coffee. Alonso-Salces et al. [60] reported that the significant difference between Arabica and Robusta coffee from different regions was the presence of caffeoyl tyrosine, which was identified only in Robusta coffee from Uganda and Angola. This is in accordance with Berti et al. [61] underlining the three types of coffee, namely, Arabica, Liberica, and Robusta, in which caffeoyl tyrosine becomes a marker compound for Robusta coffee. In addition, caffeoyl tryptophan is also identified in green beans' natural and honey processing (Table 2). Caffeoyl tryptophan was initially detected in Robusta coffee green beans [62]. However, Berti et al. [61] reported that this compound was also found in Arabica coffee, despite being lower than in Robusta coffee. According to Sullivan and Knollenberg [63], caffeoyl tyrosine and caffeoyl tryptophan are formed by the precursors hydroxycinnamoyl-CoA and phenolic amino acids (Figures 4(b) and 4(c)). However, caffeoyl tyrosine is no longer detected in roasted beans in both natural and honey processing. Meanwhile, caffeoyl tryptophan is only identified in roasted beans that are natural processed. Its concentration is less than in green beans (Table 3). That is because caffeoyl conjugate compounds are easily degraded at high temperatures [64].

The dissimilarity of the compounds between green bean samples results from various processing procedures and microbial agents. Evangelista et al. [65] reported that the bacteria identified in natural, semidry, and fully washed fermentation were various and different. *Erwinia, Klebsiella, Leuconostoc, Weissella, Enterococcus, Enterobacter, Serratia*, and *Bacillus L. mesenteroides* were identified in fully washed processing. *Erwinia* and *Klebsiella* have produced the enzyme pectinase. Besides bacteria, other microorganisms identified in fully washed processing are yeasts and filamentous fungi. *Pichia kudriavzevii, Hanseniaspora uvarum*, mesophilic aerobic bacteria (AMB) of the genus *Citrobacter, Leuconostoc mesenteroides,* and *Lactococcus lactis* produce compounds of mannitol, glycerol, and lactic acid by utilizing sugars present in mucilage [66]. Meanwhile, microorganisms identified in the natural processing including *Bacillus, Serratia, Enterobacter*, *Acinetobacter, Debaryomyces, Pichia and Candida, Aspergillus penicillium, Fusarium,* and *Cladosporium*, which showed the activity of pectinase and cellulase, which account for the production of acetic acid and lactic acid [67, 68]. In honey processing, the identified microorganisms are *Wallemia, Pichia, Kazachstania, Mortierella, Kurtzmaniella, Candida, Geotrichum, Wickerhamomyces, Meyerozyma,* and *Basidiobolus* [41].

Table 3 shows the marker compounds identified in roasted beans, including Guaiacol acetate. It is considered a marker compound in roasted beans from the natural process (Table 3). Guaiacol acetate is successfully identified in roasted beans from the natural process. It is formed through the esterification of guaiacol compounds [69]. Guaiacol is formed during roasting from the oxidation of 4-vinylguaiacol obtained from hydrolysis of 5-FQA hydrolysis and decarboxylation of ferulic acid [69]. Furthermore, 4-Ethyl-2-methyloxazole is one of the compounds only identified in roasted beans from the honey process. It was first discovered in roasted coffee beans by Vitzthum and Werkhoff [70] and is known as a Maillard reaction product [71]. In addition to these compounds, N-feruloylglycine, 3-(2-furanylmethylene)pyrrolidine, and gluconic acid are also proposed as marker compounds in roasted beans from the honey process. 3-(2-Furanylmethylene)pyrrolidine constitutes a product of the Maillard reaction between L-proline and sugar [72]. Meanwhile, gluconic acid is formed from glucose through an oxidation process during roasting [73].

Regarding 2-C-Methyl-D-erythrono-1,4-lactone, it is found in fully washed roasted beans and forms through the Amadori reaction [74]. Naringenin is a compound synthesized from the amino acid phenylalanine in the conjugated aglycon form. This compound is mainly found in oranges and tomatoes [75]. Additionally, 2-methyl-3-(methylthio)furan is also detected in roasted beans from the fully washed process and is produced via the Maillard reaction. Methylfuran originates from ribose, while methylthio is obtained from ribose and cysteine [76]. One of the benzoic acid derivatives that are only detected in the roasted beans fully washed process is 4-methoxysalicylic acid, produced from the transformation of quinic acid during roasting. The degradation of quinic acid begins with a dehydration reaction, and after this, it is oxidized to form benzoic acid [20]. In addition to 4-methoxysalicylic acid, 2-methyl-1-phenyl-2-propanyl butyrate is a benzenoid acid derivative compound that is only detected in roasted beans from a fully washed process. Besides, 2-aminoheptanedioic acid is considered a specific component in this coffee sample, which is a derivative of carboxylic acids. According to Wei and Tanokura [34], furan, carboxylic acids, and aldehydes are compound classes produced from sucrose substrates in green beans during roasting. The presence of compounds predicted as markers in each postharvest processing, and both green beans and roasted beans suggested that postharvest treatments significantly affected the composition and concentration of compounds in coffee beans. These compounds can be used as a marker for authentication during inter-postharvest processing in Arabica coffee.

## 4. Conclusion

Postharvest processing (natural, honey, and fully washed) applied to Kalosi-Enrekang Arabica coffee significantly altered the green and roasted bean's chemical composition. This present work also reported marker compounds for each processing. The number of markers in green beans for each postharvest processing differed, i.e., 3 compounds for the natural process, 6 for the honey process, and 2 for the fully washed process. Caffeoyl tyrosine was found in green beans from natural and honey processing, which was previously only identified in Robusta coffee. The markers in the roasted beans also varied between postharvest processing, i.e., 4 compounds for the natural process, 5 for the honey process, and 7 for the fully washed process. In summary, these compounds can be used to distinguish Arabica green beans from roasted coffee beans prepared by various postharvest processing methods. Further researches on how these compounds are formed need to investigate.

## Figures and Tables

**Table 1:** Compounds classes identified in green and roasted beans of Kalosi-Enrekang Arabica coffee with different postharvest processing (% relative).

Compound class	Green beans			Roasted beans		
	Natural	Honey	Fully washed	Natural	Honey	Fully washed
Quinic acid and derivative	23.76 ± 0.18^bc^^∗^	20.46 ± 0.37^a^^∗^	23.24 ± 1.92^b^^∗^	10.24 ± 0.46^a^^∗^	10.95 ± 1.33^a^^∗^	10.95 ± 1.16^a^^∗^
Cinnamic acids and derivatives	0.42 ± 0.04^c^^∗^	0.29 ± 0.03^a^^∗^	0.32 ± 0.02^ab^^∗^	1.85 ± 0.05^a^^∗^	1.63 ± 0.17^a^^∗^	1.67 ± 0.16^a^^∗^
Coumarins and derivatives	2.51 ± 0.19^a^^∗^	2.33 ± 0.29^a^	2.69 ± 0.19^a^^∗^	1.65 ± 0.28^a^^∗^	1.76 ± 0.39^a^	1.64 ± 0.27^a^^∗^
Amino acids, peptides, and analogues	7.15 ± 0.42^bc^^∗^	6.70 ± 0.29^b^^∗^	5.62 ± 0.11^a^^∗^	0.85 ± 0.07^c^^∗^	0.63 ± 0.06^b^^∗^	0.49 ± 0.05^a^^∗^
Organoheterocyclic compounds	1.49 ± 0.12^b^^∗^	1.54 ± 0.19^bc^^∗^	1.16 ± 0.05^a^^∗^	6.44 ± 0.17^bc^^∗^	6.10 ± 0.71^ab^^∗^	5.12 ± 0.52^a^^∗^
Alkaloid	26.92 ± 0.68^a^^∗^	27.70 ± 0.30^ab^	29.38 ± 1.19^bc^^∗^	24.14 ± 0.46^a^^∗^	25.10 ± 0.34^ab^	27.04 ± 1.56^bc^^∗^
Organic acids and derivatives	0.06 ± 0.00^c^^∗^	0.03 ± 0.00^a^	0.03 ± 0.00^ab^	0.03 ± 0.00^a^^∗^	0.02 ± 0.00^a^	0.03 ± 0.00^a^
Lipids and lipid-like molecules	0.41 ± 0.04^bc^	0.29 ± 0.04^a^	0.39 ± 0.04^b^^∗^	0.51 ± 0.05^a^	0.42 ± 0.08^a^	0.47 ± 0.03^a^^∗^
Phenylpropanoids and polyketides	0.11 ± 0.01^bc∗^	0.07 ± 0.01^a^^∗^	0.08 ± 0.00^ab^^∗^	nd^∗^	nd^∗^	0.004 ± 0.00^a^^∗^
Carbohydrates and carbohydrate conjugates	0.10 ± 0.01^a^^∗^	0.12 ± 0.02^ab^^∗^	0.18 ± 0.01^c^^∗^	0.47 ± 0.04^ab^^∗^	0.26 ± 0.02^a^^∗^	0.49 ± 0.03^bc^^∗^
Phenolic acids	0.10 ± 0.00^∗^	nd	nd	nd	nd	nd
Benzenoid	0.02 ± 0.00^ab^^∗^	0.03 ± 0.00^bc^^∗^	0.02 ± 0.00^a^^∗^	2.00 ± 0.28^a^^∗^	1.50 ± 0.35^a^^∗^	2.18 ± 0.50^a^^∗^
Carboxylic acids and derivatives	1.80 ± 0.11^a^^∗^	1.65 ± 0.12^a^^∗^	1.63 ± 0.05^a^^∗^	0.71 ± 0.03^c^^∗^	0.18 ± 0.02^a^^∗^	0.23 ± 0.01^ab^^∗^
Organonitrogen compounds	1.88 ± 0.23^a^	1.88 ± 0.07^a^	2.18 ± 0.17^a^	1.98 ± 0.13^a^	1.78 ± 0.26^a^	1.63 ± 0.46^a^
Purine nucleotides	0.11 ± 0.00^a^^∗^	0.14 ± 0.05^a^	0.15 ± 0.04^a^	0.13 ± 0.01^a^^∗^	0.14 ± 0.01^a^	0.13 ± 0.02^a^
Purines and purine derivatives	0.03 ± 0.00^a^^∗^	0.04 ± 0.01^a^^∗^	0.02 ± 0.00^a^^∗^	0.14 ± 0.01^a^^∗^	0.12 ± 0.00^a^^∗^	0.12 ± 0.02^a^^∗^
Pyrimidine nucleosides	0.01 ± 0.00^a∗^	0.01 ± 0.00^a^^∗^	nd	nd ^∗^	nd ^∗^	nd
Keto acids and derivatives	0.04 ± 0.00^a^	0.04 ± 0.00^a^	0.05 ± 0.00^a^	0.04 ± 0.00^a^	0.04 ± 0.00^a^	0.04 ± 0.00^a^
Carbonyl compounds	nd ^∗^	nd ^∗^	nd ^∗^	4.43 ± 0.20^a^^∗^	4.23 ± 1.00^a^^∗^	5.01 ± 0.22^a^^∗^
Pyrimidines and pyrimidine derivatives	nd ^∗^	nd ^∗^	nd ^∗^	0.03 ± 0.00^a^^∗^	0.03 ± 0.00^a^^∗^	0.03 ± 0.00^a^^∗^
Organooxygen compounds	nd ^∗^	nd ^∗^	nd ^∗^	0.44 ± 0.07^a^^∗^	0.51 ± 0.11^a^^∗^	0.44 ± 0.07^a∗^
Organosulfur compounds	nd ^∗^	nd ^∗^	nd ^∗^	0.02 ± 0.00^a^^∗^	0.01 ± 0.00^a^^∗^	0.01 ± 0.00^a^^∗^

Description: the values followed by the same superscript letter in the same row indicate no significant difference (*P* > 0.05). The value is the average % relative to the 3 replications and the standard error. The number followed by the ^∗^ sign is a significant difference between green beans and roasted beans as T-Test (*P* < 0.05). ANOVA tests on green beans and roasted beans were carried out separately.

**Table 2:** Markers compounds in green beans of Kalosi-Enrekang Arabica coffee processed by postharvest processing are different (% relative).

Compounds	Postharvest			VIP predictive
	Natural	Honey	Fully washed	
Ala-pro	0.005 ± 0.00	nd	nd	1.16
N6-acetyl-L-lysine	0.022 ± 0.00	nd	nd	1.23
Methyl caffeate	0.097 ± 0.00	nd	nd	1.22
Caffeoyl tryptophan	0.581 ± 0.03	0.663 ± 0.06	nd	1.23
Caffeoyl tyrosine	0.005 ± 0.00	0.006 ± 0.00	nd	1.23
Leucyl-phenylalanine	nd	0.004 ± 0.00	nd	1.23
Sinapoylputrescine	nd	0.008 ± 0.00	nd	1.23
1-[(3-Carboxypropyl)amino]-1-Deoxy-beta-D-fructofuranose	nd	0.016 ± 0.00	nd	1.14
D-glucuronic acid	nd	0.005 ± 0.00	nd	1.19
D-(-)-fructose	nd	0.008 ± 0.00	nd	1.18
Tyramine	nd	0.009 ± 0.00	nd	1.18
3-Hydroxycoumarin	nd	nd	0.208 ± 0.01	1.24
7-Ethoxycoumarin	nd	nd	0.006 ± 0.00	1.24

Description: value is the average % relative and the standard deviation of 3 replications. nd: undetectable.

**Table 3:** Marker compounds in roasted beans of Kalosi-Enrekang Arabica coffee processed by postharvest processing are different (% relative).

Compounds	Postharvest			VIP predictive
	Natural	Honey	Fully washed	
Caffeoyl tryptophan	0.227 ± 0.05	nd	nd	1.42
L-valine	0.020 ± 0.00	nd	nd	1.45
Guaiacol acetate	0.029 ± 0.00	nd	nd	1.42
Indole-3-acetyl-valine	0.019 ± 0.00	nd	nd	1.44
Coriandrone E	0.003 ± 0.00	nd	nd	1.44
Picrocrocin	0.005 ± 0.00	nd	nd	1.44
N-feruloylglycine	nd	0.007 ± 0.00	nd	1.35
3-(2-Furanylmethylene)pyrrolidine	nd	0.050 ± 0.01	nd	1.38
4-Ethyl-2-methyloxazole	nd	0.012 ± 0.00	nd	1.44
Gluconic acid	nd	0.012 ± 0.00	nd	1.40
2-Acetyl-6-methylpyridine	nd	0.004 ± 0.00	nd	1.31
2-C-methyl-D-erythrono-1,4-lactone	nd	nd	0.008 ± 0.00	1.40
Homoarecoline	nd	nd	0.007 ± 0.00	1.40
Naringenin	nd	nd	0.004 ± 0.00	1.41
2-Methyl-1-phenyl-2-propanyl butyrate	nd	nd	0.004 ± 0.00	1.41
4-Methoxysalicylic acid	nd	nd	0.048 ± 0.00	1.40
2-Aminoheptanedioic acid	nd	nd	0.005 ± 0.00	1.26
2-Methyl-3-(methylthio)furan	nd	nd	0.003 ± 0.00	1.17

Description: the value is the average % relative and standard deviation of 3 replications. nd: undetectable.

## Data Availability

The data obtained and analyzed from this study are included in the article and the supplementary materials.

## Supplementary Materials

Supplementary Materials The supplementary material consists of Table 1, Figure 1, and Figure 2. Table 1 contains the classes of compounds and compounds identified in green beans and roasted beans of Kalosi-Enrekang Arabica coffee prepared with different postharvest processing and analyzed using LC-MS/MS. Meanwhile, Figure 1 contains the VIP-predictive score of compounds identified in green and roasted beans. This is used to validate the selected compounds as markers in postharvest processing. Compounds with a score of VIP> 1.00 significantly affect the separation/differentiation during postharvest processing. Figure 2 contains the results of permutation tests on green beans and roasted beans prepared with different postharvest processing methods. The permutation test was used to validate the feasibility of OPLS-DA modeling in distinguishing postharvest processing applied to Kalosi-Enrekang Arabica coffee.
