# Supplementary material for: Detection of Markers in Green Beans and Roasted Beans of Kalosi-Enrekang Arabica Coffee with Different Postharvest Processing Using LC-MS/MS
Source: Int J Food Sci. 2023 Mar 23;2023:6696808. doi: 10.1155/2023/6696808 (PMC10063361; doi:10.1155/2023/6696808)
Supplement: Supplementary Materials — The supplementary material consists of Table 1, Figure 1, and Figure 2. Table 1 contains the classes of compounds and compounds identified in green beans and roasted beans of Kalosi-Enrekang Arabica coffee prepared with different postharvest processing and analyzed using LC-MS/MS. Meanwhile, Figure 1 contains the VIP-predictive score of compounds identified in green and roasted beans. This is used to validate the selected compounds as markers in postharvest processing. Compounds with a score of VIP> 1.00 significantly affect the separation/differentiation during postharvest processing. Figure 2 contains the results of permutation tests on green beans and roasted beans prepared with different postharvest processing methods. The permutation test was used to validate the feasibility of OPLS-DA modeling in distinguishing postharvest processing applied to Kalosi-Enrekang Arabica coffee. [file 6696808.f1.pdf]

Supplementary materials contain about identified of compounds in green beans and roasted beans Kalosi-Enrekang Arabica (% area relative) (Table 1), VIP predictive OPLS-DA modeling of identified compounds in green beans (A) and green beans (B) (Figure 1), and permutation test of green beans (A) dan roasted beans (B) (Figure 2).

Table 1 contains the classes of compounds and compounds identified in green beans and roasted beans Kalosi-Enrekang Arabica prepared with different postharvest processing and analyzed using LC-MS/MS.

Table 1. Identified of compounds in green beans and roasted beans Arabica Kalosi Enrekang (%relative)

Compounds	Formula	Eror ppm	MW	RT	MS^2	M/Z	Postharvest					
							Green beans			Roasted beans		
							Natural	Honey	Fully washed	Natural	Honey	Fully washed
**Quinic Acid and Derivative**												
5 Feruloylquinic acid	C17 H20 O9	-2.59	368.10978	8.147	M+H	369.1163	3.550	3.073	3.223	1.801	1.719	1.751
3 Feruloylquinic acid	C17 H20 O9	-2.29	368.10989	6.644	M+H	369.1163	0.153	0.109	0.123	0.187	0.188	0.179
4 Feruloylquinic acid	C17 H20 O9	-2.34	368.10987	9.105	M+H	369.1163	0.036	0.055	0.043	0.008	0.008	0.006
5 Caffeoylquinic acids	C16 H18 O9	-2.83	354.09408	6.359	M+H	355.1017	13.590	9.141	11.607	4.603	4.771	4.221
4 Caffeoylquinic acids	C16 H18 O9	-2.61	354.09416	6.819	M+H	355.1017	2.707	3.744	4.003	1.947	2.377	2.327
3 Caffeoylquinic acids	C16 H18 O9	-3.16	354.09396	4.983	M+H	355.1017	1.492	2.315	2.200	0.933	0.983	1.762
D-(-)-Quinic acid	C7 H12 O6	-4.92	192.06244	1.102	M+H	193.0341	0.236	0.247	0.236	0.016	0.091	0.014
4-O-p-coumaroylquinic acid	C16 H18 O8	-2.55	338.09931	7.631	M+H	339.1066	0.632	0.459	0.544	0.269	0.227	0.227
5-O-p-coumaroylquinic acid	C16 H18 O8	-1.28	338.09973	5.96	M+H	339.1068	0.025	0.016	0.018	0.025	0.024	0.025
3-O-p-coumaroylquinic acid	C16 H18 O8	-1.08	338.0998	5.785	M+H	339.1067	0.021	0.012	0.009	0.021	0.010	0.010
3,5-Dicaffeoylquinic acid	C25 H24 O12	-2.13	516.12568	9.83	M-H	515.1187	0.715	0.738	0.697	0.217	0.295	0.216
4,5-Dicaffeoylquinic acid	C25 H24 O12	-2.18	516.12565	10.701	M-H	515.1187	0.597	0.548	0.536	0.175	0.238	0.171
3,4-Dicaffeoylquinic acid	C25 H24 O12	-1.88	516.12581	8.302	M-H	515.1186	nd	nd	nd	0.031	0.023	0.029
1,4-Di-O-caffeoylquinic acid	C25 H24 O12	-1.82	516.12584	7.146	M+H	517.1332	nd	nd	nd	0.011	nd	0.011
**Total**							**23.752**	**20.456**	**23.240**	**10.244**	**10.953**	**10.949**
**Cinnamic acids and derivatives**												
4-Coumaroyl-2-hydroxyputrescine	C13 H18 N2 O3	-1.79	250.1313	7.459	M+H	251.1639	0.014	0.009	0.014	nd	nd	nd
(E)-p-coumaric acid	C9 H8 O3	-0.56	164.04725	1.74	M+H	165.0606	0.012	0.058	0.012	0.025	0.016	0.027
(E)-Ferulic acid	C10 H10 O4	-1.97	194.05753	4.985	M-H	193.0498	0.050	0.013	0.077	0.080	0.078	0.081
alpha-Terpinyl cinnamate	C19 H24 O2	-2.03	284.17705	9.685	M+H	285.1837	0.018	0.015	0.015	0.117	0.111	0.098

Compounds	Formula	Eror ppm	MW	RT	MS^2	M/Z	Postharvest					
							Green beans			Roasted beans		
							Natural	Honey	Fully washed	Natural	Honey	Fully washed
Caffeic acid	C9 H8 O4	-4.28	180.04149	6.953	M-H	179.0840	0.171	0.095	0.087	0.017	0.013	0.014
Cinnamic acid	C9 H8 O2	-1.19	148.05225	8.135	M+H	149.0595	0.025	0.022	0.022	0.026	0.024	0.025
5-Hydroxyferulic acid	C10 H10 O5	-3.05	210.05218	7.887	M+H	211.0824	0.017	0.017	nd	nd	nd	nd
Ferulic acid	C10 H10 O4	-3.87	194.05716	10.544	M-H	193.0496	0.011	0.009	0.013	0.113	0.086	0.110
4-Coumaroylputrescine	C13 H18 N2 O2	-2.56	234.13623	5.759	M+H	235.1689	0.050	0.039	0.043	nd	nd	nd
Sinapoylputrescine	C15 H22 N2 O4	-1.43	294.15754	3.508	M+H	295.1644	nd	0.008	nd	nd	0.009	0.006
Sinapic acid	C11 H12 O5	-2.14	224.06799	7.382	M+H	225.0753	nd	nd	nd	0.019	0.015	0.016
2-O-(E-p-Hydroxycinnamoyl)-(x)-glyceric acid	C12 H12 O6	-2.9	252.06266	10.657	M+H	253.0698	nd	nd	nd	0.063	0.051	0.066
N-trans-p-Coumaroyloctopamine	C17 H17 N O4	-2.51	299.11501	7.208	M+H	300.1225	nd	nd	nd	0.011	0.008	nd
caffeoylshikimate	C16 H16 O8	-2.69	336.08361	7.939	M-H	335.0766	nd	nd	nd	0.688	0.610	0.673
3-O-Caffeoylshikimic acid	C16 H16 O8	-2.35	336.08373	8.481	M-H	335.0766	nd	nd	nd	0.449	0.401	0.452
4-O-Caffeoylshikimic acid	C16 H16 O8	-3.63	336.0833	8.124	M-H	335.0767	nd	nd	nd	0.162	0.122	nd
3-Coumaric acid	C9 H8 O3	-6.16	164.04633	7.149	M-H	163.0391	nd	nd	nd	0.010	0.008	nd
(E)-4-Methoxycinnamic acid	C10 H10 O3	-2.58	178.06253	8.373	M-H	177.0549	nd	nd	nd	0.011	0.006	0.027
(E)-3,4,5-Trimethoxycinnamic acid	C12 H14 O5	-2.85	238.08344	8.877	M+H	239.0883	nd	nd	nd	0.013	0.013	0.012
4-coumaroylshikimic acid	C16 H16 O7	-1.56	320.0891	8.994	M-H	319.0819	nd	nd	nd	nd	0.012	0.017
1-O-2'-Hydroxy-4'-methoxycinnamoyl-b-D-glucose	C16 H20 O9	-1.93	356.11004	6.526	M+H	357.2027	nd	nd	nd	nd	nd	nd
3,4-Dimethoxy-cinnamic acid	C11 H12 O4	-1.78	208.07319	8.243	M+H	209.0805	0.055	0.004	0.038	0.052	0.045	0.043
**Total**							**0.424**	**0.288**	**0.321**	**1.855**	**1.629**	**1.668**
**Coumarins and derivatives**												
4-Hydroxy-2H-1-benzopyran-2-one	C9 H6 O3	-2.87	162.03123	6.38	M+H	163.0384	1.719	1.657	1.771	0.944	1.160	0.951
Coumarin	C9 H6 O2	-2.4	146.03643	7.639	M+H	147.0438	0.092	0.070	0.086	0.054	0.048	0.055
8-methoxycoumarin	C10 H8 O3	-3.43	176.04674	8.155	M+H	177.0540	0.700	0.600	0.623	0.440	0.389	0.426
3 Hydroxycoumarin	C9 H6 O3	-3.12	162.03119	4.993	M+H	163.0385	nd	nd	0.208	0.074	0.067	0.076
7-Ethoxycoumarin	C11 H10 O3	-1.41	190.06273	12.504	M+H	191.0701	nd	nd	0.006	nd	nd	nd
Scopoletin	C10 H8 O4	-2.03	192.04187	7.442	M+H	193.0491	nd	nd	nd	0.060	0.042	0.053
Scoparone	C11 H10 O4	-4.03	206.05708	12.918	M+H	207.0643	nd	nd	nd	0.070	0.045	0.065

Compounds	Formula	Eror ppm	MW	RT	MS^2	M/Z	Postharvest					
							Green beans			Roasted beans		
							Natural	Honey	Fully washed	Natural	Honey	Fully washed
7-Ethoxy-4-methyl-2H-1-benzopyran-2-one	C12 H12 O3	-3.91	204.07785	8.686	M+H	205.0858	nd	nd	nd	0.009	0.006	0.010
4-Methylumbelliferyl acetate	C12 H10 O4	-2.54	218.05736	11.181	M-H	217.0500	nd	nd	nd	nd	0.007	0.009
**Total**							**2.511**	**2.327**	**2.694**	**1.651**	**1.763**	**1.644**
**Amino acids, peptides, and analogues**												
N-feruloylglycine	C12 H13 N O5	-1.71	251.07894	6.998	M-H	250.0715	0.045	0.038	0.046	nd	0.007	nd
N-Acetyl-L-phenylalanine	C11 H13 N O3	-1.56	207.08922	6.818	M+H	208.0962	0.025	0.027	0.024	0.032	0.030	0.032
N-Acetyl-L-leucine	C8 H15 N O3	-2.57	173.10475	8.359	M+H	174.1122	0.016	0.013	0.174	0.018	0.014	0.005
Na-p-Hydroxycoumaroyltryptophan	C20 H18 N2 O4	-3.39	350.12547	12.356	M+H	351.1324	0.212	0.215	0.456	0.072	0.092	0.061
N-Caffeoyltryptophan	C20 H18 N2 O5	-3.33	366.12035	11.646	M+H	367.1273	0.581	0.663	nd	0.227	nd	nd
Leucylproline	C11 H20 N2 O3	-1.74	228.147	5.64	M+H	229.1803	0.048	0.042	nd	nd	nd	nd
Leu-Leu	C12 H24 N2 O3	-1.6	244.1783	7.321	M+H	245.0781	0.006	0.007	nd	nd	nd	nd
L-Valine	C5 H11 N O2	-1.15	117.07884	1.195	M+H	118.0863	0.391	0.336	0.292	0.020	nd	nd
L-Tyrosine	C9 H11 N O3	-2.77	181.07339	1.725	M+H	182.0806	0.162	0.141	0.130	0.172	0.193	0.138
L-Theanine	C7 H14 N2 O3	-0.88	174.10029	1.154	M+H	175.0863	0.019	0.023	0.016	nd	nd	nd
L-Pyroglutamic acid	C5 H7 N O3	-0.88	129.04248	1.564	M-H	128.0343	0.013	0.011	0.011	nd	0.040	nd
L-Phenylalanine	C9 H11 N O2	-2.53	165.07856	3.356	M+H	166.0858	1.498	1.047	1.293	0.070	0.055	0.048
L-Isoleucine	C6 H13 N O2	-1.95	131.09437	1.866	M+H	132.1017	1.630	1.629	1.112	nd	nd	nd
L-(-)-Methionine	C5 H11 N O2 S	-2.25	149.05071	1.346	M+H	150.1123	0.141	0.109	0.065	nd	nd	nd
L-(-)-Asparagine	C4 H8 N2 O3	-0.94	132.05337	1.188	M-H	133.0131	0.033	0.053	0.043	nd	nd	nd
L-(+)-Arginine	C6 H14 N4 O2	-1.31	174.11145	1.151	M+H	174.9753	0.065	0.064	0.050	nd	nd	nd
L-(+)-Alanine	C3 H7 N O2	2.72	89.04792	1.171	M+H	90.0916	0.057	0.060	0.084	nd	nd	nd
gamma-Aminobutyric acid	C4 H9 N O2	0.72	103.0634	1.108	M+H	104.0344	0.750	0.767	0.469	nd	nd	nd
DL-Lysine	C6 H14 N2 O2	-1.33	146.10533	1.13	M-H	147.0653	0.041	nd	nd	nd	nd	nd
DL-Histidine	C6 H9 N3 O2	-1.09	155.06931	1.167	M+H	155.1062	0.023	0.026	0.025	nd	nd	nd
DL-Glutamic acid	C5 H9 N O4	-2.21	147.05283	1.175	M-H	146.9821	0.100	0.107	0.188	nd	nd	nd
D-(+)-Tryptophan	C11 H12 N2 O2	-2.86	204.08929	5.09	M+H	205.0968	0.815	0.860	0.823	0.010	0.018	0.011
D-(+)-Proline	C5 H9 N O2	-0.27	115.0633	1.138	M+H	116.0706	0.383	0.373	0.298	nd	nd	nd
Ala-Pro	C8 H14 N2 O3	-2.09	186.10005	1.166	M+H	187.0077	0.005	nd	nd	nd	nd	nd

Compounds	Formula	Eror ppm	MW	RT	MS^2	M/Z	Postharvest					
							Green beans			Roasted beans		
							Natural	Honey	Fully washed	Natural	Honey	Fully washed
Leucyl-phenylalanine	C15 H22 N2 O3	-0.99	278.16277	9.06	M+H	279.1589	nd	0.004	nd	nd	nd	nd
L-arogenate	C10 H13 N O5	-1.35	227.07907	7.91	M+H	228.2319	0.018	0.016	0.014	nd	nd	nd
L-Isoleucyl-L-proline	C11 H20 N2 O3	-2.03	228.14693	1.172	M+H	229.0542	nd	0.007	0.008	nd	nd	nd
Cyclo(leucylprolyl)	C11 H18 N2 O2	-1.16	210.13658	8.855	M+H	211.0824	0.024	0.014	nd	nd	nd	nd
1-[(3-Carboxypropyl)amino]-1-deoxy-beta-D-fructofuranose	C10 H19 N O7	-0.4	265.11604	1.111	M-H	264.0874	nd	0.016	nd	nd	nd	nd
N-(1-Deoxy-1-fructosyl)leucine	C12 H23 N O7	-0.95	293.14717	2.154	M+H	294.2058	0.046	0.023	nd	nd	nd	nd
Z-Leu-OH	C14 H19 N O4	-2.72	265.13069	4.878	M+H	266.1380	nd	nd	nd	0.043	0.033	0.038
N-Benzoylaspartic acid	C11 H11 N O5	-2.06	237.06323	4.184	M+H	238.0703	nd	nd	nd	0.009	nd	0.019
N-Acetylvanilalanine	C12 H15 N O5	-2.55	253.09438	5.871	M+H	254.1017	nd	nd	nd	0.088	0.065	0.076
N-Acetyl-L-cysteine	C5 H9 N O3 S	-2.66	163.02988	6.691	M+H	164.0372	nd	nd	nd	0.027	0.029	0.024
N-Acetyl-DL-tryptophan	C13 H14 N2 O3	-2.21	246.0999	9.191	M+H	247.1072	nd	nd	nd	0.026	0.030	0.029
(3xi,6xi)-Cyclo(alanylvalyl)	C8 H14 N2 O2	-1.57	170.10526	2.025	M+H	171.1125	nd	nd	nd	0.012	0.011	nd
L-(+)-Cysteine	C3 H7 N O2 S	-0.78	121.01966	6.695	M+H	122.0269	nd	nd	nd	0.008	0.008	0.007
Indole-3-acetyl-valine	C15 H18 N2 O3	-2.48	274.13106	5.974	M-H	273.0338	nd	nd	nd	0.019	nd	nd
2-Aminoheptanedioic acid	C7 H13 N O4	-1.65	175.08417	4.544	M+H	176.0103	nd	nd	nd	nd	nd	0.005
Caffeoyl tyrosine	C18 H17 N O6	-1.99	343.10491	9.31	M+H	344.2248	0.005	0.006	nd	nd	nd	nd
**Total**							**7.151**	**6.696**	**5.622**	**0.853**	**0.625**	**0.494**
**Organoheterocyclic compounds**												
Tryptamine	C10 H12 N2	-2.8	160.0996	5.478	M+H	161.0594	0.069	0.147	0.028	nd	nd	nd
Serotonin	C10 H12 N2 O	-2.96	176.09444	2.631	M+H	177.1019	0.095	0.099	0.048	0.024	0.022	0.013
trans-Zeatin	C10 H13 N5 O	-3.6	219.11122	1.868	M+H	220.1184	0.306	0.345	0.318	0.213	0.258	0.211
1,3,7-Trimethyluric acid	C8 H10 N4 O3	-1.53	210.07497	7.261	M+H	211.0824	0.055	nd	0.053	nd	nd	nd
Phthalide	C8 H6 O2	-1.3	134.0366	6.386	M+H	135.0438	0.092	0.088	0.108	0.044	0.039	0.045
Guanine	C5 H5 N5 O	-1.96	151.04911	1.357	M+H	152.0561	0.015	0.028	0.019	nd	0.024	0.019
Nicotinic acid	C6 H5 N O2	-0.73	123.03194	1.416	M+H	124.0391	0.023	0.021	nd	0.241	0.203	0.232
6-Aminonicotinamide	C6 H7 N3 O	-1.79	137.05867	7.11	M+H	138.0659	0.041	nd	0.041	0.037	0.044	0.048
Pyridoxine	C8 H11 N O3	-1.87	169.07358	1.226	M+H	170.0808	nd	nd	nd	0.053	0.058	0.027
Pyridoxine phosphate	C8 H12 N O6 P	-1.84	249.03977	7.315	M+H	150.0470	nd	nd	nd	0.008	0.009	nd

Compounds	Formula	Eror ppm	MW	RT	MS^2	M/Z	Postharvest					
							Green beans			Roasted beans		
							Natural	Honey	Fully washed	Natural	Honey	Fully washed
Pyridine	C5 H5 N	3.78	79.0425	1.269	M+H	80.0497	nd	nd	nd	0.381	0.250	0.426
3-Propylpyridine	C8 H11 N	-0.86	121.08905	1.174	M+H	122.0964	nd	nd	nd	0.016	0.012	0.022
Nepetalactam	C10 H15 N O	-2.39	165.11497	5.951	M+H	166.1222	nd	nd	nd	0.066	0.044	0.058
Nicotinamide	C6 H6 N2 O	-0.97	122.04789	1.638	M+H	123.0552	nd	nd	nd	0.039	0.037	nd
2-Ethyl-5-methylpyridine	C8 H11 N	-0.87	121.08904	1.758	M+H	122.0963	nd	nd	nd	0.092	0.060	0.108
2-Methylpyridine	C6 H7 N	1.68	93.05801	1.112	M+H	94.0653	nd	nd	nd	1.170	0.899	nd
Methyl nicotinate	C7 H7 N O2	-3.12	137.04725	6.241	M+H	138.0545	nd	nd	nd	0.101	0.105	0.115
5-Acetyl-2,4-dimethyloxazole	C7 H9 N O2	-2.65	139.06296	1.103	M+H	140.0701	0.019	0.018	0.021	0.123	0.157	nd
5-Acetyl-2,4-dimethylthiazole	C7 H9 N O S	-2.47	155.0401	3.873	M+H	156.1381	nd	nd	nd	0.008	nd	0.007
5-Methyl-2-propyloxazole	C7 H11 N O	-0.87	125.08396	8.612	M+H	126.0913	nd	nd	nd	0.020	0.016	0.009
4-Ethyl-2-methyloxazole	C6 H9 N O	-0.2	111.06839	3.437	M+H	112.0757	nd	nd	nd	nd	0.012	nd
gamma-Butyrolactone	C4 H6 O2	3.33	86.03707	1.108	M+H	87.0442	0.106	0.121	0.074	0.716	0.522	0.534
2-C-Methyl-1,4-erythrono-D-lactone	C5 H8 O4	-1.66	132.04204	1.287	M+H	133.0343	nd	nd	nd	nd	nd	0.008
6-Methylquinoline	C10 H9 N	-2.59	143.07313	5.525	M+H	144.1015	0.097	0.200	0.040	nd	nd	nd
6-Methoxyquinoline	C10 H9 N O	-2.79	159.06797	2.63	M+H	160.0752	0.090	0.099	0.050	nd	nd	nd
N-hydroxy-L-tryptophan	C11 H12 N2 O3	-1.88	220.08438	2.308	M-H	218.9817	0.022	0.026	0.027	nd	nd	nd
Indole-3-glycol	C10 H11 N O2	-2.6	177.07852	11.073	M+H	178.0860	nd	nd	nd	0.011	0.010	0.011
N-acetyl serotonin	C12 H14 N2 O2	-2.32	218.10502	9.722	M+H	219.1122	nd	nd	nd	0.008	0.013	0.008
Tryptophol	C10 H11 N O	-2	161.08374	12.105	M+H	162.0910	nd	nd	nd	0.003	0.003	nd
Methyl dioxindole-3-acetate	C11 H11 N O4	-2.46	221.06826	7.552	M+H	222.0755	nd	nd	nd	0.026	0.019	0.031
Indole-3-acetic acid	C10 H9 N O2	-3.26	175.06276	3.816	M+H	176.0701	nd	nd	nd	0.107	0.075	0.122
Indole-3-acetate	C10 H9 N O2	-3.27	175.06276	6.078	M+H	176.0702	nd	nd	nd	0.046	0.039	0.057
Mascaroside	C26 H36 O10	-4.34	508.22864	10.151	M+H	509.2356	0.456	0.347	0.330	nd	0.110	0.080
Cafestol	C20 H28 O3	-2.66	316.203	16.401	M+H	317.2103	nd	nd	nd	0.007	0.007	0.006
Tetramethylpyrazine	C8 H12 N2	-2.32	136.09973	10.515	M+H	137.1070	nd	nd	nd	0.043	0.045	0.042
2-Isopropyl-5-methoxypyrazine	C8 H12 N2 O	-2.85	152.09453	6.501	M+H	153.1018	nd	nd	nd	0.082	0.113	0.075
2-Isopropyl-3,5-dimethoxy-6-methylpyrazine	C10 H16 N2 O2	-2.46	196.1207	6.676	M+H	197.1279	nd	nd	nd	0.045	0.050	0.049
6,7-Dihydro-2-methyl-5H-cyclopenta[b]pyrazine	C8 H10 N2	-1.52	134.08419	9.046	M+H	135.0915	nd	nd	nd	0.012	0.014	0.014

Compounds	Formula	Eror ppm	MW	RT	MS^2	M/Z	Postharvest					
							Green beans			Roasted beans		
							Natural	Honey	Fully washed	Natural	Honey	Fully washed
1-Propenylpyrazine	C7 H8 N2	-0.46	120.06869	7.325	M+H	121.0760	nd	nd	nd	0.009	0.011	0.009
3-Ethyl-2-methoxypyrazine	C7 H10 N2 O	-2.37	138.07899	4.969	M+H	139.0861	nd	nd	nd	0.119	0.128	0.110
2-Methoxy-3-(2-methylpropyl)pyrazine	C9 H14 N2 O	-2.02	166.11028	8.469	M+H	167.1176	nd	nd	nd	0.015	0.013	0.014
2,6-Diemthyl-pyrazine	C6 H8 N2	-0.53	108.06869	6.715	M+H	109.0760	nd	nd	nd	0.344	0.364	0.346
5-Methyl-2-ethyl-pyrazine	C7 H10 N2	-1.46	122.08422	9.071	M+H	123.0915	nd	nd	nd	0.104	0.102	0.099
6-Methyl-2-ethyl-pyrazine	C7 H10 N2	-1.46	122.08422	8.03	M+H	123.0915	nd	nd	nd	0.069	0.076	0.061
Methylpyrazine	C5 H6 N2	1.31	94.05322	4.782	M+H	95.0605	nd	nd	nd	0.395	0.447	0.398
xi-Salsolinol	C10 H13 N O2	-1.73	179.09432	1.642	M+H	180.1016	nd	nd	nd	0.025	0.012	0.028
Propyl 2-furoate	C8 H10 O3	-2.8	154.06256	10.738	M+H	155.0699	nd	nd	nd	0.015	0.017	0.015
Ethyl 2-phenyl-3-furancarboxylate	C13 H12 O3	-1.51	216.07832	9.053	M+H	217.0856	nd	nd	nd	0.003	nd	0.003
Furfuryl acetate	C7 H8 O3	-2.92	140.04694	8.26	M+H	141.0542	nd	nd	nd	0.076	0.082	0.111
2,5-Furandicarboxylic acid	C6 H4 O5	-1.58	156.00563	1.499	M+H	157.0128	nd	nd	nd	0.012	0.012	0.011
3-(2-Furanylmethylene)pyrrolidine	C9 H11 N O	-2.39	149.08371	1.16	M+H	150.1122	nd	nd	nd	nd	0.050	nd
Ethyl maltol	C7 H8 O3	-2.93	140.04693	5.086	M+H	141.0543	nd	nd	nd	0.073	0.055	0.062
Kojic acid	C6 H6 O4	-2.29	142.02628	4.818	M+H	143.0335	nd	nd	nd	0.082	0.111	0.085
4-Hydroxy-2-butenoic acid gamma-lactone	C4 H4 O2	2.85	84.02137	1.716	M+H	85.0285	nd	nd	nd	0.191	0.204	0.205
Norfuraneol	C5 H6 O3	-0.92	114.03159	2.722	M+H	115.0389	nd	nd	nd	0.207	0.196	0.193
3-Hydroxy-4,5-dimethyl-2(5H)-furanone	C6 H8 O3	-2.2	128.04706	5.087	M+H	129.0543	nd	nd	nd	0.586	0.650	0.595
Pratenol A	C14 H12 O5	-1.8	260.06801	9.719	M-H	259.0608	nd	nd	nd	0.005	0.010	0.012
Eugenitin	C12 H12 O4	-2.4	220.07303	9.424	M+H	221.0804	nd	nd	nd	0.009	0.010	0.010
Coriandrone E	C13 H12 O5	-2.19	248.06793	9.616	M-H	247.0607	nd	nd	nd	0.003	nd	nd
Dillapional	C12 H12 O5	-2.45	236.06789	5.857	M+H	235.0606	nd	nd	nd	0.116	0.103	0.109
4-Amino-2-methyl-5-phosphomethylpyrimidine	C6 H10 N3 O4 P	3.13	219.04158	7.972	M+H	220.0488	nd	nd	nd	0.014	0.015	0.015
Clarycet	C11 H20 O3	-2.17	200.14081	14.865	M+H	201.1481	nd	nd	0.004	0.006	0.002	nd
Methylisopelletierine	C9 H17 N O	-1.72	155.13075	12.303	M+H	156.1380	nd	nd	nd	0.005	0.003	0.016
Isosalsolidine	C12 H13 N O2	-3.55	203.09391	2.46	M+H	204.1011	nd	nd	nd	0.178	0.138	0.190

Compounds	Formula	Eror ppm	MW	RT	MS^2	M/Z	Postharvest					
							Green beans			Roasted beans		
							Natural	Honey	Fully washed	Natural	Honey	Fully washed
2,3-Dihydro-5-(5-methyl-2-furanyl)-1H-pyrrolizine	C12 H13 N O	-1.71	187.09939	5.88	M+H	188.1066	nd	nd	nd	0.023	0.014	0.038
3-Oxo-carbofuran	C12 H13 N O4	-2.14	235.08396	9.299	M+H	236.0913	nd	nd	nd	0.012	0.007	0.010
**Total**							**1.486**	**1.539**	**1.162**	**6.438**	**6.101**	**5.123**
**Alkaloid**												
Trigonelline	C7 H7 N O2	-2.91	137.04728	1.119	M+H	138.0544	10.858	10.740	12.014	9.687	10.243	10.347
Theobromine	C7 H8 N4 O2	-1.75	180.06441	4.72	M+H	181.0717	0.022	0.025	0.022	0.019	0.029	0.022
Theophyline	C7 H8 N4 O2	-1.85	180.06439	4.529	M+H	181.0717	0.012	0.007	0.010	nd	0.007	0.009
Caffeine	C8 H10 N4 O2	-3.78	194.07964	7.106	M+H	195.0867	15.839	16.784	17.148	14.362	14.768	16.617
3beta,6beta-Dihydroxynortropane	C7 H13 N O2	-2.04	143.09434	1.206	M+H	144.0806	0.189	0.146	0.191	nd	nd	nd
Arecoline	C8 H13 N O2	-2.37	155.09426	5.553	M+H	156.1016	nd	nd	nd	0.062	0.043	0.029
Hygrine	C8 H15 N O	-2.36	141.11503	1.404	M+H	142.1224	nd	nd	nd	0.011	0.013	0.010
Homoarecoline	C9 H15 N O2	-2.23	169.1099	6.892	M+H	170.0807	nd	nd	nd	nd	nd	0.007
**Total**							**26.920**	**27.702**	**29.385**	**24.142**	**25.104**	**27.041**
**Organic acids and derivatives**												
Triethyl citrate	C12 H20 O7	-1.95	276.12037	12.151	M-H	275.1132	0.034	0.025	0.030	0.031	0.022	0.025
N6-Acetyl-L-lysine	C8 H16 N2 O3	-1.36	188.11584	1.19	M+H	188.0700	0.022	nd	nd	nd	nd	nd
**Total**							**0.056**	**0.025**	**0.030**	**0.031**	**0.022**	**0.025**
**Lipids and lipid-like molecules**												
Stearic acid	C18 H36 O2	-0.74	284.27132	22.979	M-H	283.2638	0.032	0.027	0.039	0.031	0.040	0.033
Palmitelaidic acid	C16 H30 O2	-2.19	254.22402	20.366	M+H	253.1277	0.010	nd	nd	nd	nd	nd
alpha-Linolenic acid	C18 H30 O2	-1.05	278.22429	21.112	M-H	277.2169	0.004	nd	0.003	nd	nd	nd
Oleic acid	C18 H34 O2	-1.32	282.25551	19.323	M-H	281.1391	0.007	nd	nd	nd	nd	nd
Linolenelaidic acid	C18 H30 O2	-1.96	278.22403	18.921	M-H	277.2169	0.011	0.006	0.009	nd	nd	nd
Linoleic acid	C18 H32 O2	-1.17	280.2399	22.126	M-H	279.2324	0.058	0.027	0.064	0.011	0.006	0.013
Stearidonic acid	C18 H28 O2	-1.4	276.20854	18.391	M+H	277.2131	0.004	nd	0.003	nd	nd	nd
1-Linoleoyl glycerol	C21 H38 O4	-1.85	354.27635	20.994	M+H	355.1012	0.004	0.002	nd	nd	nd	nd
Traumatic Acid	C12 H20 O4	-1.02	228.13593	10.943	M+H	229.1404	nd	nd	0.003	nd	nd	nd
8-Hydroxyhexadecanedioic acid	C16 H30 O5	-2.48	302.20857	17.04	M+H	303.2157	nd	nd	0.005	0.012	0.004	0.002

Compounds	Formula	Eror ppm	MW	RT	MS^2	M/Z	Postharvest					
							Green beans			Roasted beans		
							Natural	Honey	Fully washed	Natural	Honey	Fully washed
(-)-pinellic acid	C18 H34 O5	-3.97	330.23931	18.678	M+H	313.1768	nd	nd	0.024	0.056	0.020	0.009
Sorbic acid	C6 H8 O2	-0.68	112.05235	5.991	M+H	113.0596	nd	nd	nd	0.202	0.149	0.209
Prostaglandin B1	C20 H32 O4	-1.74	336.22948	16.082	M-H	335.2222	nd	nd	nd	0.004	nd	0.004
Diethyl maleate	C8 H12 O4	-1.19	172.07335	5.283	M+H	173.0807	nd	nd	nd	0.020	nd	0.018
Prostaglandin B-2	C20 H30 O4	-1.55	334.21389	14.368	M-H	333.2066	nd	nd	nd	0.030	0.025	0.028
g-Butyrobetaine	C7 H15 N O2	-2.09	145.10998	1.129	M+H	146.1172	nd	nd	nd	0.050	0.035	0.042
Tetradecanoylcarnitine	C21 H41 N O4	-2.67	371.30257	21	M+H	372.1280	nd	nd	nd	0.012	nd	nd
11-Oxooctadecanoic acid	C18 H34 O3	-2.21	298.25014	19.193	M-H	297.2429	nd	nd	nd	nd	0.006	nd
Stearoyllactic acid	C21 H40 O4	-2.6	356.29173	22.05	M+H	357.2029	nd	nd	nd	nd	0.001	nd
(Z)-3-(1-Formyl-1-propenyl)pentanedioic acid	C9 H12 O5	-2.24	200.06803	4.405	M+H	201.0753	nd	nd	nd	nd	0.038	0.031
Butenylcarnitine	C11 H19 N O4	-2.93	229.13074	13.856	M+H	230.2472	nd	nd	nd	nd	nd	0.003
Palmitic acid	C16 H32 O2	-1.72	256.23979	23.263	M+H	274.2726	nd	nd	nd	nd	nd	nd
Gibberellin A24	C20 H26 O5	-4.29	346.17654	9.687	M+H	347.1847	0.175	0.147	0.149	nd	nd	nd
Gibberellin A12	C20 H28 O4	-1.84	332.19815	9.364	M+H	333.2052	0.009	nd	0.009	0.013	0.014	0.013
Crocetin	C20 H24 O4	-2.48	328.16665	9.686	M+H	329.1740	0.032	0.026	0.026	nd	nd	nd
Atractyligenin	C19 H28 O4	-1.57	320.19826	11.754	M-H	319.1910	nd	nd	nd	0.008	0.005	0.007
alpha-Crocetin glucosyl ester	C26 H34 O9	-2.74	490.21894	13.168	M+H	491.2262	nd	nd	nd	0.039	0.039	0.030
Kahweol	C20 H26 O3	-2.57	314.18739	13.253	M+H	315.1947	nd	nd	nd	0.011	0.012	0.012
(+/-)-Camphor	C10 H16 O	-1.69	152.11986	8.149	M+H	153.0546	0.013	0.010	0.008	nd	nd	nd
Citroside	C19 H30 O8	-1.21	386.1936	8.018	M-H	385.1133	0.011	0.010	0.008	nd	nd	nd
L-alpha-Glycerylphosphorylcholine	C8 H20 N O6 P	-2.02	257.1023	1.173	M+H	258.1095	0.032	0.039	0.037	0.015	0.025	0.019
1-Stearoylglycerol	C21 H42 O4	-1.92	358.30762	20.038	M+H	359.3150	0.011	nd	nd	nd	nd	nd
Total							**0.414**	**0.293**	**0.386**	**0.512**	**0.418**	**0.472**
**Phenylpropanoids and polyketides**												
Naringenin	C15 H12 O5	-2.71	272.06774	8.631	M-H	271.2273	nd	nd	nd	nd	nd	0.004
Prunin	C21 H22 O10	-1.82	434.12051	9.771	M+H	435.1279	nd	nd	nd	nd	nd	nd
Rhaponticin	C21 H24 O9	-2.48	420.14099	11.326	M+H	421.1485	0.109	0.074	0.084	nd	nd	nd
**Total**							**0.109**	**0.074**	**0.084**	**nd**	**nd**	**0.004**

Compounds	Formula	Eror ppm	MW	RT	MS^2	M/Z	Postharvest					
							Green beans			Roasted beans		
							Natural	Honey	Fully washed	Natural	Honey	Fully washed
**Carbohydrates and carbohydrate conjugates**												
Picrocrocin	C16 H26 O7	-1.71	330.16729	15.569	M+H	331.1742	0.018	0.004	0.015	0.005	nd	nd
Glucoheptonic Acid	C7 H14 O8	-2.81	226.06823	1.047	M-H	225.0609	0.013	nd	0.008	0.014	0.019	nd
D-(-)-Mannitol	C6 H14 O6	-4.85	182.07815	1.055	M+H	183.1014	0.011	0.006	0.008	nd	nd	nd
D-(-)-3-Phosphoglyceric acid	C3 H7 O7 P	-0.9	185.99277	1.816	M+H	187.0078	0.006	0.087	0.103	nd	nd	nd
5-Aminoimidazole ribonucleotide	C8 H14 N3 O7 P	4.11	295.05815	1.033	M+H	296.0655	0.035	nd	0.035	nd	nd	nd
4-Hydroxybenzoic acid glucoside	C13 H16 O8	-1.08	300.08419	7.079	M-H	299.2225	0.012	0.008	0.010	nd	nd	nd
D-Glucuronic acid	C6 H10 O7	-4.59	194.04176	1.058	M+H	195.0871	nd	0.005	nd	nd	nd	nd
D-(-)-Fructose	C6 H12 O6	-4.62	180.06256	1.067	M+H	181.9869	nd	0.008	nd	nd	nd	nd
ribulose 5-phosphate	C5 H11 O8 P	-1.92	230.01871	1.13	M+H	231.0259	nd	nd	nd	0.266	0.229	0.289
Erythrose, 4-phosphate	C4 H9 O7 P	-1.71	200.00825	1.152	M+H	201.0156	nd	nd	nd	0.186	nd	0.205
Gluconic acid	C6 H12 O7	-5.74	196.05718	1.077	M+H	196.0905	nd	nd	nd	nd	0.012	nd
Total							**0.095**	**0.118**	**0.178**	**0.470**	**0.260**	**0.494**
**Phenolic acids**												
Methyl caffeate	C10 H10 O4	-1.92	194.05754	6.824	M+H	195.0870	0.097	nd	nd	nd	nd	nd
**Total**							**0.097**	**nd**	**nd**	**nd**	**nd**	**nd**
**Benzenoid**												
2-Phenylpropyl isobutyrate	C13 H18 O2	-1.43	206.13038	7.993	M+H	207.1375	0.021	0.021	0.017	0.014	0.015	0.013
Tyramine	C8 H11 N O	-0.96	137.08393	1.867	M+H	138.0911	nd	0.009	nd	0.023	0.017	0.023
Salicylic acid	C7 H6 O3	-1.76	138.03145	5.337	M+H	139.0387	nd	nd	nd	0.013	0.015	0.013
Propylparaben	C10 H12 O3	-1.39	180.07839	10.065	M+H	181.0856	nd	nd	nd	0.008	nd	0.008
Zein	C7 H7 F O2 S	1.42	174.01533	1.155	M-H	173.0080	nd	nd	nd	0.022	0.020	0.019
1-nitro-2-phenylethane	C8 H9 N O2	-3.19	151.06285	1.571	M+H	152.0702	nd	nd	nd	0.121	0.111	0.118
Eudesmic acid	C10 H12 O5	-3.37	212.06776	9.649	M-H	211.0605	nd	nd	nd	0.013	0.010	0.013
3-Aminosalicylic acid	C7 H7 N O3	-3.97	153.04199	6.948	M+H	154.0493	nd	nd	nd	0.137	0.159	0.129
N-Methyltyramine	C9 H13 N O	-2.13	151.09939	4.093	M+H	152.1066	nd	nd	nd	0.040	0.027	0.035
Salicyaldehyde	C13 H12 N2 O	-2.33	212.09447	8.486	M+H	213.1017	nd	nd	nd	0.016	0.022	0.015
(R)-Bitalin A	C13 H14 O3	-2.4	218.09377	11.016	M+H	219.1011	nd	nd	nd	0.013	0.006	0.014
Allyl phenoxyacetate	C11 H12 O3	-3.11	192.07805	10.254	M+H	193.0858	nd	nd	nd	0.011	0.008	0.012

Compounds	Formula	Eror ppm	MW	RT	MS^2	M/Z	Postharvest					
							Green beans			Roasted beans		
							Natural	Honey	Fully washed	Natural	Honey	Fully washed
Allyl anthranilate	C10 H11 N O2	-2.58	177.07852	6.891	M+H	178.0858	nd	nd	nd	0.037	0.044	0.029
2-(3-Phenylpropyl)tetrahydrofuran	C13 H18 O	-2.17	190.13535	16.179	M+H	191.0701	nd	nd	nd	0.005	nd	0.004
Methyl anthranilate	C8 H9 N O2	-3.79	151.06276	8.897	M+H	152.0701	nd	nd	nd	0.082	0.066	nd
Methyl N-methylanthranilate	C9 H11 N O2	-3.03	165.07848	1.94	M+H	166.0860	nd	nd	nd	0.054	nd	0.039
Ethyl anthranilate	C9 H11 N O2	-3.06	165.07847	10.231	M-H	181.0497	nd	nd	nd	0.024	0.018	nd
methyl 4-hydroxy-3-methoxybenzoate	C9 H10 O4	-1	182.05773	9.545	M+H	183.0914	nd	nd	nd	nd	0.004	0.004
2-Methyl-1-phenyl-2-propanyl butyrate	C14 H20 O2	-1.72	220.14595	8.943	M+H	211.1435	nd	nd	nd	nd	nd	0.004
4-Methoxysalicylic acid	C8 H8 O4	-3.71	168.04163	7.316	M+H	169.0856	nd	nd	nd	nd	nd	0.048
2-Methoxy-4-(4-methyl-1,3-dioxolan-2-yl)phenol	C11 H14 O4	-3.72	210.08843	7.102	M-H	209.0812	nd	nd	nd	0.056	0.051	0.056
Phloroglucinol	C6 H6 O3	-2.51	126.03138	4.836	M+H	127.0388	nd	nd	nd	0.697	0.358	0.863
1-(3-Hydroxy-4-methoxyphenyl)-1,2-ethanediol	C9 H12 O4	-1.87	184.07321	6.368	M+H	185.0918	nd	nd	nd	0.034	0.034	0.041
1,2-Benzenediol	C6 H6 O2	-0.94	110.03668	6.83	M+H	111.0440	nd	nd	nd	0.320	0.265	0.344
1,3-Benzenediol	C6 H6 O2	-0.91	110.03668	3.321	M+H	111.0441	nd	nd	nd	0.046	0.064	0.068
2,6-Dimethoxy-4-(1-propenyl)phenol	C11 H14 O3	-4.62	194.0934	10.267	M-H	193.0861	nd	nd	nd	0.005	0.004	0.005
(R)-Synephrine	C9 H13 N O3	-1.74	183.08923	4.74	M+H	184.0965	nd	nd	nd	0.020	0.019	0.016
Vanillyl alcohol	C8 H10 O3	-3.1	154.06252	4.841	M+H	155.0699	nd	nd	nd	nd	0.039	0.050
Toluidine	C7 H9 N	0.54	107.07356	1.118	M+H	108.0808	nd	nd	nd	0.049	0.032	0.050
4-Amino-2-methyl-1-naphthol	C11 H11 N O	-1.99	173.08372	3.909	M+H	174.0910	nd	nd	nd	0.066	0.055	0.102
4-Methoxy-1-naphthol	C11 H10 O2	-1.72	174.06778	12.194	M+H	175.0750	nd	nd	nd	0.004	0.004	0.004
Juglone	C10 H6 O3	-1.57	174.03142	8.754	M+H	175.0387	nd	nd	nd	0.010	0.007	nd
1,4-dihydroxy-2-naphthoate	C11 H8 O4	-2.12	204.04183	9.252	M+H	205.0856	nd	nd	nd	0.010	0.011	0.011
Vanillin isobutyrate	C12 H14 O4	-1.99	222.08877	13.111	M+H	223.0961	nd	nd	nd	0.009	0.006	0.008
Guaicyl acetate	C9 H10 O3	-4.63	166.06222	9.384	M+H	167.0700	nd	nd	nd	0.029	nd	nd
1'-Acetoxyeugenol acetate	C14 H16 O5	-2.23	264.09918	9.725	M+H	265.1065	nd	nd	nd	0.009	0.007	0.013
Isoeugenol formate	C11 H12 O3	-1.67	192.07832	7.931	M+H	193.0858	nd	nd	nd	0.005	0.004	0.005
**Total**							**0.021**	**0.031**	**0.017**	**2.003**	**1.505**	**2.178**

Compounds	Formula	Eror ppm	MW	RT	MS^2	M/Z	Postharvest					
							Green beans			Roasted beans		
							Natural	Honey	Fully washed	Natural	Honey	Fully washed
**Carboxylic acids and derivatives**												
Propyl propane thiosulfonate	C11 H16 O5	-1.75	228.09938	11.882	M+H	229.1065	0.007	0.007	0.009	0.006	0.007	0.007
Citric acid	C6 H8 O7	-4.97	192.02605	1.172	M-H	191.0186	1.762	1.614	1.592	0.534	nd	nd
Capsi-amide	C17 H35 N O	-1.8	269.27138	21.613	M-H	268.1466	0.002	nd	nd	nd	nd	nd
€-Aconitic acid	C6 H6 O6	-1.73	174.01614	1.494	M+H	175.0234	0.029	0.033	0.033	0.026	0.027	0.030
2-succinylbenzoate	C11 H10 O5	-3.76	222.05199	9.255	M+H	223.0593	nd	nd	nd	0.052	0.053	nd
Ginsenoyne H	C19 H26 O3	-4.48	302.18684	10.17	M+H	303.1947	nd	nd	nd	0.096	0.092	0.085
Ethyl pyruvate	C5 H8 O3	-1.05	116.04722	1.598	M+H	117.0545	nd	nd	nd	nd	nd	0.108
Total							**1.799**	**1.654**	**1.634**	**0.714**	**0.179**	**0.229**
**Organonitrogen compounds**												
Choline	C5 H13 N O	0.88	103.0998	1.089	M+H	104.1069	1.836	1.837	2.181	1.979	1.776	1.632
Putrescine	C4 H12 N2	3.23	88.1004	1.074			0.042	0.039	nd	nd	nd	nd
Total							**1.877**	**1.877**	**2.181**	**1.979**	**1.776**	**1.632**
**Purine nucleotides**												
Adenosine 5'-monophosphate	C10 H14 N5 O7 P	-1.64	347.06252	1.33	M+H	348.2165	0.006	0.007	0.005	nd	nd	nd
Adenosine	C10 H13 N5 O4	-3.26	267.09588	2.675	M+H	268.1029	0.106	0.131	0.140	0.133	0.136	0.126
**Total**							**0.112**	**0.138**	**0.145**	**0.133**	**0.136**	**0.126**
**Purines and purine derivatives**												
Adenine	C5 H5 N5	-2.86	135.05411	1.355	M+H	136.0614	0.028	0.042	0.021	0.138	0.116	0.122
**Total**							**0.028**	**0.042**	**0.021**	**0.138**	**0.116**	**0.122**
**Pyrimidine nucleosides**												
Cytidine	C9 H13 N3 O5	-1	243.08528	1.291	M+H	244.2269	0.009	0.010	nd	nd	nd	nd
**Total**							**0.009**	**0.010**	**nd**	**nd**	**nd**	**nd**
**Keto acids and derivatives**												
alpha-Ketoglutaric acid	C5 H6 O5	-2.28	146.02119	1.493	M+H	147.0285	0.037	0.040	0.047	0.039	0.042	0.044
**Total**							**0.037**	**0.040**	**0.047**	**0.039**	**0.042**	**0.044**
**Carbonyl compounds**												
Furfural	C5 H4 O2	2.37	96.02136	11.878	M+H	97.0285	nd	nd	nd	0.091	0.082	0.095
Isomaltol	C6 H6 O3	-2.54	126.03137	3.476	M+H	127.0386	nd	nd	nd	2.056	1.919	2.294

Compounds	Formula	Eror ppm	MW	RT	MS^2	M/Z	Postharvest					
							Green beans			Roasted beans		
							Natural	Honey	Fully washed	Natural	Honey	Fully washed
Pyrrol-2-methylketone	C6 H7 N O	-0.51	109.05271	1.46	M+H	110.0599	nd	nd	nd	0.663	0.597	0.637
4'-Methoxyacetophenone	C9 H10 O2	-1.92	150.06779	10.145	M+H	151.0751	nd	nd	nd	0.008	0.004	0.007
1H-Pyrrole-2-carboxaldehyde	C5 H5 N O	0.89	95.0372	1.185	M+H	96.0445	nd	nd	nd	1.550	1.450	1.795
4-Acetyl-2-methylpyrimidine	C7 H8 N2 O	-2	136.06339	7.414	M+H	137.0706	nd	nd	nd	0.039	0.046	0.038
1-(5-Methyl-2-furanyl)-1,2-propanedione	C8 H8 O3	-7.42	152.04622	7.32	M-H	151.0390	nd	nd	nd	nd	0.014	0.009
2'-Hydroxyacetophenone	C8 H8 O2	-1.58	136.05221	7.877	M+H	137.0595	nd	nd	nd	nd	0.017	0.016
3-Methyl-1,2-cyclohexanedione	C7 H10 O2	-1.4	126.0679	7.949	M-H	125.0232	nd	nd	nd	nd	0.062	0.084
3,5-Dimethyl-1,2-cyclopentanedione	C7 H10 O2	-1.34	126.06791	7.927	M+H	127.0378	nd	nd	nd	nd	nd	nd
3,4-Dimethyl-1,2-cyclopentanedione	C7 H10 O2	-1.34	126.06791	7.673	M+H	127.0378	nd	nd	nd	nd	nd	nd
gamma-Oxo-3-pyridinebutanal	C9 H9 N O2	-2.45	163.06293	5.814	M+H	164.0702	nd	nd	nd	0.026	0.034	0.039
**Total**							nd	nd	**nd**	**4.433**	**4.227**	**5.014**
**Pyrimidines and pyrimidine derivatives**												
Pyrimidine	C4 H4 N2	3.56	80.03773	2.66	M+H	81.0450	nd	nd	nd	0.020	0.024	0.021
Uracil	C4 H4 N2 O2	0.14	112.02729	1.724	M+H	113.0346	nd	nd	nd	0.010	0.009	0.009
Total							**nd**	**nd**	**nd**	**0.030**	**0.033**	**0.030**
**Organooxygen compounds**												
2-Acetyl-3,6-dimethylpyrazine	C8 H10 N2 O	-1.84	150.07904	6.09	M+H	151.0863	nd	nd	nd	0.009	0.008	0.008
2-Acetyl-3,5-dimethylpyrazine	C8 H10 N2 O	-1.84	150.07904	6.941	M+H	151.0864	nd	nd	nd	0.007	0.008	0.007
2-Acetyl-3-ethylpyrazine	C8 H10 N2 O	-1.6	150.07907	9.561	M+H	151.0864	nd	nd	nd	0.004	0.005	nd
2-Acetyloxazole	C5 H5 N O2	-0.29	111.032	1.572	M+H	112.0393	nd	nd	nd	0.026	0.023	0.028
benzoquinone	C6 H4 O2	-0.33	108.02109	3.433	M+H	109.0284	nd	nd	nd	0.328	0.345	0.281
4-Acetyl-2-methylpyridine	C8 H9 N O	-2.46	135.06808	1.485	M+H	136.0755	nd	nd	nd	0.038	0.034	0.037
4-Acetyl-3-methylpyridine	C8 H9 N O	-2.47	135.06808	8.836	M+H	136.0754	nd	nd	nd	0.021	0.020	0.012
2-Acetyl-5-methylpyridine	C8 H9 N O	-2.45	135.06808	10.382	M+H	136.0754	nd	nd	nd	0.005	0.005	0.005
2-Acetyl-6-methylpyridine	C8 H9 N O	-2.08	135.06813	9.934	M+H	136.0755	nd	nd	nd	nd	0.004	nd
Ethyl 2-furanyl diketone	C8 H8 O3	-4.32	152.04669	6.549	M-H	151.0390	nd	nd	nd	nd	0.056	0.064
**Total**							**nd**	**nd**	**nd**	**0.440**	**0.507**	**0.443**
**Organosulfur compounds**												

Compounds	Formula	Eror ppm	MW	RT	MS^2	M/Z	Postharvest					
							Green beans			Roasted beans		
							Natural	Honey	Fully washed	Natural	Honey	Fully washed
Kahweofuran	C5 H7 N O	1.25	97.05288	8.917	M+H	98.0602	nd	nd	nd	0.015	0.014	0.010
2-Methyl-3-(methylthio)furan	C6 H8 O S	-1.41	128.02941	7.626	M+H	129.0521	nd	nd	nd	nd	nd	0.003
**Total**							**nd**	**nd**	**nd**	**0.015**	**0.014**	**0.013**

Description: nd: not detected; MW: molecular weight; RT: Retention time; MS^2: Mass spectrometry (ion positive and negative); M/Z: mass to charge ratio.

Figure 1 contains VIP-predictive score of compounds identified in green beans and roasted beans. This is used to validate the selected compounds as markers in postharvest processing. Compounds have a score VIP>1.0 significantly affect the separation/differentiation of postharvest processing.

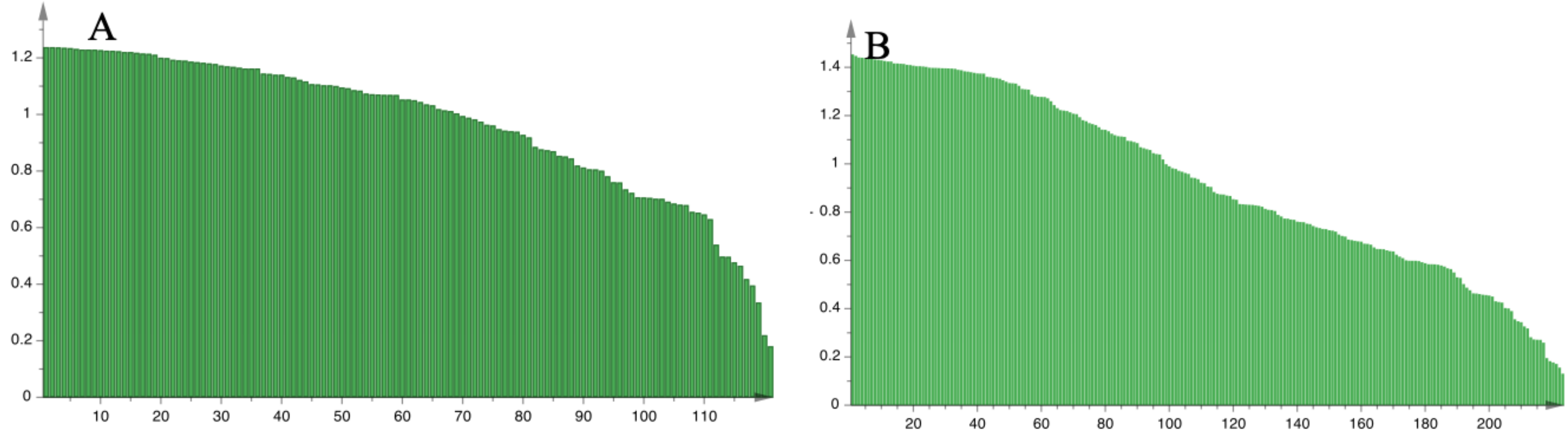

Figure 1. VIP Predictive OPLS-DA modeling of compounds identified in green beans (A) and roasted beans (B)

Figure 2 contains the results of permutation tests on green beans and roasted beans prepared with different postharvest processing. The permutation test was used to validate the feasibility of OPLS-DA modeling in distinguishing postharvest processing applied to Kalosi-Enrekang Arabica coffee.

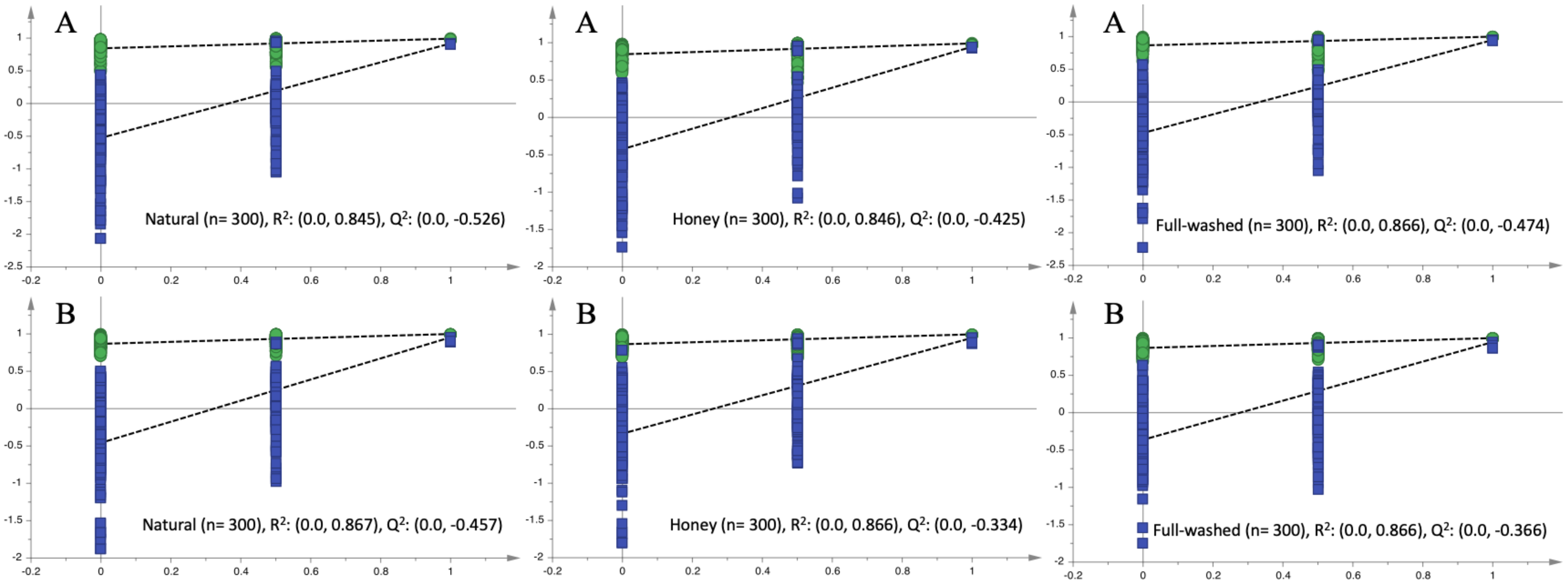

Figure 2. Permutation Test of Green Beans (A) dan Roasted Beans (B)
